# New discoveries of the family Epicopeiidae from China, with description of a new species (Lepidoptera, Epicopeiidae)

**DOI:** 10.3897/zookeys.822.32341

**Published:** 2019-02-04

**Authors:** Si-Yao Huang, Min Wang, Wa Da, Xiao-Ling Fan

**Affiliations:** 1 Department of Entomology, College of Agriculture, South China Agricultural University, Guangzhou 510642, Guangdong, China South China Agricultural University Guangzhou China; 2 Tibet Plateau Institute of Biology, Lhasa 850001, Xizang Autonomous Prefecture, China Tibet Plateau Institute of Biology Lhasa China

**Keywords:** Geometroidea, Himalaya, Indochina, Oriental swallowtail moth, taxonomy

## Abstract

Some new discoveries of the family Epicopeiidae Swinhoe, 1892 from China are reported. A new species, *Mimaporiaowadai* Huang & Wang, **sp. n.** is described from W. Sichuan. *Burmeia* Minet, 2003 and *Psychostrophiaendoi* Inoue, 1992 are reported as new to China, with the female genitalia of the former described for the first time. The females of *Psychostrophiaendoi* Inoue, 1992 and *Deuveiabanghaasi* Hering, 1932 are reported for the first time. Adults and genitalia of all species aforementioned are illustrated.

## Introduction

The family Epicopeiidae Swinhoe, 1892 is a small group belonging to Geometroidea hitherto comprised of ten genera and approximately 25 species restricted in the Asian Palaearctic and Oriental regions (Wei and Yen 2017). They are well known as mimics of many other lepidopterous families such as Papilionidae, Riodinidae, Pieridae, Nymphalidae, Zygaenidae, Geometridae, and Erebidae (Wei and Yen 2015). Members of this family usually share the following autapomorphies: head without ocelli, forewing without an areole and in forewing venation vein R_5_ usually stalked with vein M_1_ (Minet 2003). Nearly all of the species in Epicopeiidae are day-flying creatures, with the exception of *Amanaangulifera* Walker, 1855, which is nocturnal and can be attracted to light in the night (Wei and Yen 2015). Early stage is poorly known in this family, but in a few cases that have already been revealed, the larva and pupa are mainly covered by waxy matter on the surface (Janet and Wytsman 1903, Sugi 1972, Nakamura 2006).

**Figure 1.**
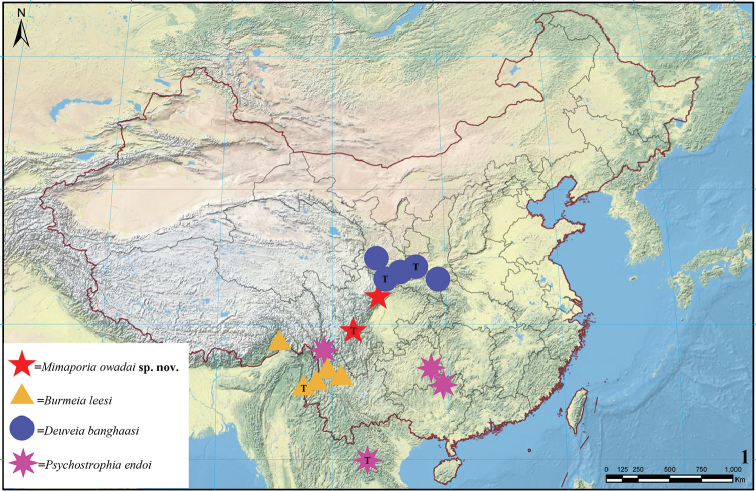
Distribution of part of the Epicopeiidae species in China. The T refers to the type locality of each species. Records of distribution are taken from Zhu et al. (2004), Wei and Yen (2017), Hering (1932), Minet (2003), and results of this work.

In mainland China, this family is poorly studied. Most of the genera included in Epicopeiidae in modern concept were placed and studied in Epipleminae (formerly Epiplemidae, now regarded as a subfamily of Uraniidae) in Chinese literatures, except for the genus *Epicopeia* Westwood, 1844. In Zhu (1981), three genera and three species placed in Epiplemidae and one genus, five species, and two subspecies placed in Epicopeiidae are all belonging to the Epicopeiidae in modern concept. In Zhu et al. (2004), three genera and nine species of Epicopeiidae have been recorded (all in Epiplemidae). In the present study, nine genera and 22 species of Epicopeiidae are recorded in mainland China, and most of them are distributed in rather high altitudes in the mountainous region of western China (Fig. 1). During the research of the lepidopterous collection of South China Agricultural University, we found a male belonging to a previously undescribed species of the genus *Mimaporia* Wei & Yen, 2017, which has already been figured in Wei and Yen (2017). The examination of the genitalia proves it to be a distinct species of this genus and is described herein. Meanwhile, female individuals of three little known species, viz. *Burmeialeesi* Minet, 2003, *Psychostrophiaendoi* Inoue, 1992, and *Deuveiabanghaasi* (Hering, 1932) are also found in the collection and female genitalia of these three species are illustrated for the first time. Among them, *Burmeialeesi* Minet and *Psychostrophiaendoi* Inoue are recorded for the first time in China.

## Materials and methods

Specimens examined in this study were all collected in daytime by an insect net, and are deposited in the collection of South China Agricultural University (SCAU), Guangzhou. All adult photos were taken by a Nikon CoolPix S7000 camera, the adults in wild and habitat photos are taken by Sony DSC-TX100 and Sony DSC-RX100 v1.00 cameras. Abdomens were removed and macerated in 10% KOH for examination of male and female genitalia. Photographs of the male genitalia of *Mimaporiaowadai* sp. n. were taken under a Zeiss SteReo Discovery V.12 digital microscope, and genitalia photos of other species were all taken under a Keyence VHX-5000 digital microscope. Adult and genitalia photos were all processed by Adobe Photoshop CS5 software. Terminology of adult and genitalia follows Klots (1970), Kristensen (2003), Minet (2003), and Wei and Yen (2017). New records are indicated by asterisk (*).

## Taxonomy

### Mimaporia

Taxon classificationAnimaliaLepidopteraEpicopeiidae

Wei & Yen, 2017

Mimaporia Wei & Yen, 2017: 542, type species: Mimaporiahmong Wei & Yen, 2017.

#### Diagnosis.

The genus *Mimaporia* is characterized by the following characters: chaetosemata absent, forewing vein M_2_ situated closer to vein M_3_ than to the stem of vein R_5_ and M_1_ and aedeagus with a strongly sclerotized shaft.

### Mimaporia owadai

Taxon classificationAnimaliaLepidopteraEpicopeiidae

Huang & Wang sp. n.

http://zoobank.org/73A9A812-E80B-4CF8-BC60-770D04D113FE

Figs 2–4 , 5–8 , 9

Mimaporia sp.: Wei & Yen, 2017, 544, 547, fig. 11.

#### Type material.

**Holotype**: male, altitude 2800 m, 3.VIII.2004, Moxi Town, Luding County, Ganzi Tibetan Autonomous Prefecture, PR China, leg. Min Wang (SCAU).

#### Diagnosis.

Externally, male of *Mimaporiaowadai* sp. n. is similar to that of the type species of the genus, *M.hmong* Wei & Yen, 2017, but it can be immediately distinguished from the latter externally by the bipectinate antenna, more yellowish patterns on upper side of both wings, hindwing upper side with a well-developed grayish basal zone and with submarginal band weakly developed, with only trails in cell M_1_ to cell CuA_2_. In male genitalia, *Mimaporiaowadai* sp. n. can be distinguished easily from *M.hmong* by the tip of the valve obviously narrowing and ending with a sharper apex, praesacculus ending with a single-branched process, juxta with a median triangular process directing ventrally and with the sclerotized shaft in aedeagus narrowing in the middle part. The individual figured in Wei and Yen (2017: 544, fig. 11) is identical with the holotype in external features, which should be regarded as the same species.

#### Description.

**Figures 2–4.**
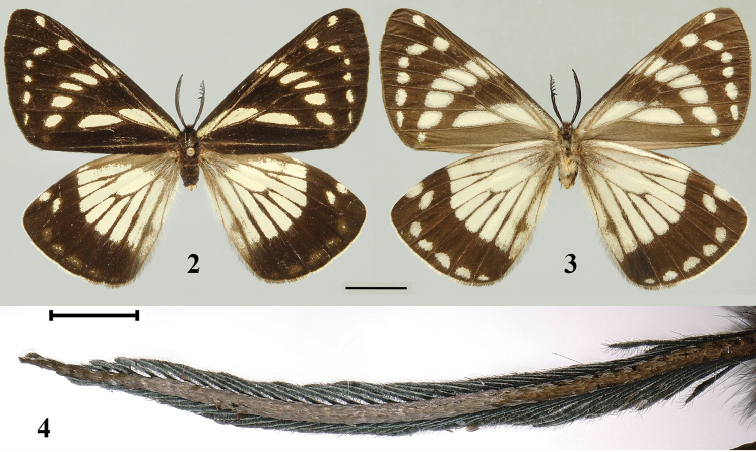
*Mimaporiaowadai* sp. n., adult: **2** upperside **3** underside **4** enlarged antenna. Scale bars: 1 cm (**2, 3**); 1 mm (**4**).

Male (Figs 2–4). Length of forewing 35 mm, wingspan 62 mm. Head black; frons wide, covered with long blackish brown hairy scales and white scales; vertex covered with long blackish brown hair; compound eye black and large; antenna black, bipectinate. Thorax black; patagia covered with white scale and long blackish brown hairy scales; tegula covered with long blackish brown hairy scales and brownish scales, and surrounded by short white scales; abdomen covered with black scales and long blackish brown hair dorsally and yellowish white hair ventrally. Forewing upper side ground color black, all patterns creamy white, cilia black. Costa with a slender stripe at base; a triangle cell bar present at the base of discoidal cell; median band consisted of six patches of different sizes and shape extending from costa to cell CuA_1_; postmedian band consisted of six dots of different sizes and shape extending from cell R_4_ to cell CuA_1_ and shifting outwards at cell R_5_ and M_1_; a single rounded dot present in cell R_3_ at subapical zone. Forewing under side ground color blackish brown, paler at basal area; patterns similar to upper side. Hindwing upper side ground color black, all patterns creamy white, cilia yellowish white from apex to vein CuA_1_, and gradually become blackish brown from vein CuA_1_ to anal angle. Wing base and about half of the hindwing costa grayish brown; median zone with a single large patch extending from costa to dorsum, and are divided into smaller and slender patches by darkened veins. Two dots present at subapical zone; submarginal band poorly-developed, with only trail of paler patches extending from cell m_1_ to cell CuA_2_. Hindwing underside ground color similar to forewing underside, submarginal band well-developed and represented by five separated yellowish white patches extending from cell M_1_ to CuA_2_; other patterns similar to the upper side.

**Figures 5–8.**
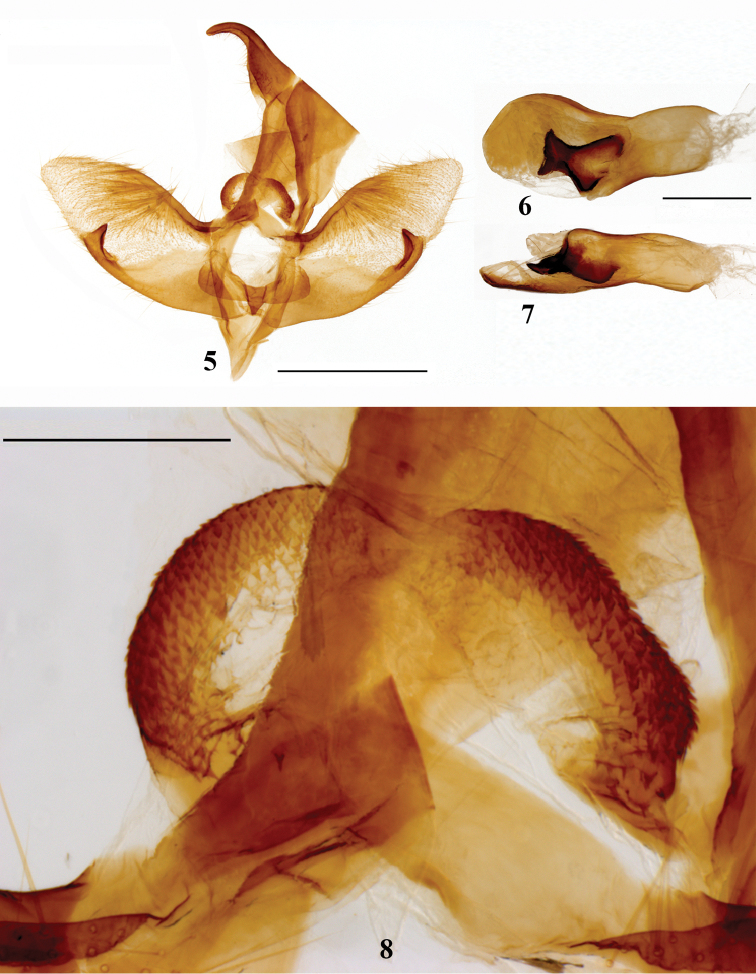
Male genitalia of *Mimaporiaowadai* sp. n.: **5** genitalia capsule ventral view **6** aedeagus dorsal view **7** aedeagus lateral view **8** enlarged area of genitalia capsule showing costula. Scale bar: 1 mm.

**Male genitalia** (Figs 5–8). Uncus well developed, bending downwards at distal one third. Tegumen broad. Costula (Fig. 8) presents at the junction of tegumen and vinculum, semi-circular and concaves shallowly at the middle, its surface scobinated by numerous small spines at both side of marginal area of the concave portion. Transtilla broad, slightly sclerotized. Juxta broad, U-shaped, with a triangular process at the middle part. Saccus narrow, triangular. Valva broad, gradually narrowing towards apex and ending with a rounded tip; its inner surface densely setose. Costa strongly sclerotized. Sacculus sclerotized, broad at base and narrowing towards tip. Praesacculus strongly sclerotized, ending with a triangular process directed dorsally. Aedeagus short and stout, narrowing at the middle and expanding near the apex in dorsal view. A strongly pigmented and sclerotized shaft present in the aedeagus, with middle part narrowing.

**Female.** Unknown.

#### Distribution.

This species is currently known to occur in Luding and Wenchuan counties (Wolong) in western and northwestern Sichuan province at present.

#### Etymology.

The specific name *owadai* is named in honor of Dr. Mamoru Owada (Tsukuba, Japan) who provided us with assistance and some literature.

#### Remarks.

**Figure 9.**
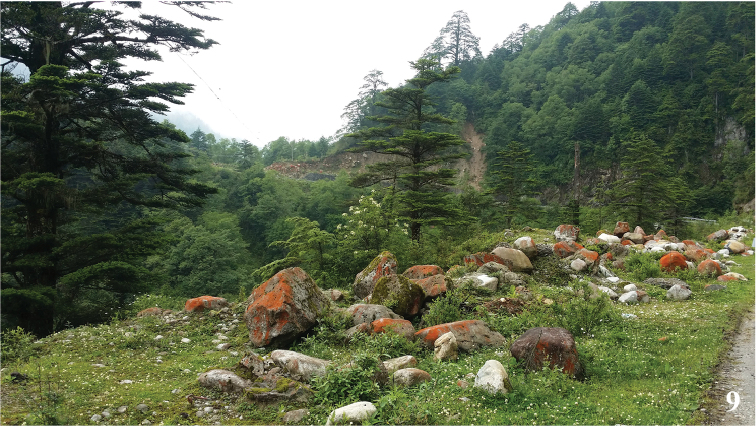
Habitat of *Mimaporiaowadai* sp. n. in Moxi County.

This new species flew like a *Neptis* (Nymphalidae) butterfly in conifer-broadleaf forest (Fig. 9) at the altitude about 2800 m. Actually, the wing maculation of forewing of the genus *Mimaporia* is also similar to certain species in the genus *Neptis*, for example *Neptisalwina* (Bremer & Grey, 1852), *Neptisdejeani* Oberthür, 1894 and *Neptisphilyroides* Staudinger, 1887, by upper side sharing similar median and postmedian band and cell bar on forewing as well as postmedian band on hindwing. The distribution area of *Mimaporiaowadai* sp. n. is within the distribution area of the former two species, while distribution area of *M.hmong* (northern Vietnam) falls within the distribution area of the last species. So we suspect that the genus *Mimaporia* might probably also a mimicker of the genus *Neptis* Fabricius, 1807 (Nymphalidae).

### Burmeia

Taxon classificationAnimaliaLepidopteraEpicopeiidae

Minet, 2003

Burmeia Minet, 2003: 470, type species: Burmeialeesi Minet, 2003.

#### Diagnosis.

The genus *Burmeia* is a monotypic genus characterized by the following characters: antennal flagellum of male without scale, hindwing termen obviously angulate between vein M_2_ and vein M_3_ and in male genitalia tegumen and vinculum synscleritous. The female genitalia of *Burmeia* are also diagnostic and they differ from the ground plan of that of *Psychostrophia* in following aspects (female genitalia of other species in the genus *Psychostrophia* have been figured by Zhu et al. 2004): antrum membranous, while sclerotized in *Psychostrophia*; ductus bursae sclerotized, while membranous in *Psychostrophia*; corpus bursae nearly the same size of the 8^th^ abdominal segment, while much larger in *Psychostrophia*; corpus bursae with an appendix bursae of similar size, while without an appendix bursae in *Psychostrophia*. Regarding the characters of female genitalia given above, *Burmeia* is definitely a distinct genus from *Psychostrophia*.

### Burmeia leesi

Taxon classificationAnimaliaLepidopteraEpicopeiidae

Minet, 2003

Figs 10–11 , 12–15 , 16, 17 , 18, 19 , 20–24

Burmeia leesi Minet, 2003: 473, fig. 2, 8, 10, 18–23.
Psychostrophia nymphidiaria : Zhu et al. 2004: 222, fig. 154, pl. VI, fig. 1 [misidentification].

#### Material examined.

28 males, 1 female, altitude 2700 m, 18–21.VII.2017, 62K, Motuo County, Linzhi Division, Xizang Autonomous Region, leg. Si-yao Huang & Shu-qin Ji; 1 male, altitude 2500 m, 15.VII.2018, Yaojiaping, Lushui County, Nujiang Lisu Autonomous Prefecture, Yunnan Province, leg. Si-yao Huang; 1 male, altitude 3000 m, 16.VII.2016, Yulong Naxi Autonomous County, Lijiang City, Yunnan Province, leg. Si-yao Huang.

#### Diagnosis.

*Burmeialeesi* Minet is unique among the Epicopeiidae by the morphological characters mentioned above in the generic diagnosis. The female genitalia are recorded here for the first time and the description is given below. The male genitalia (Figs 12–15) have already been described precisely by Minet (2003).

**Figures 10, 11.**
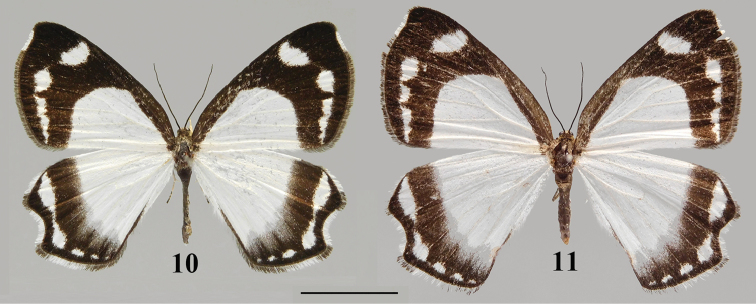
*Burmeialeesi* adult: **10** male, Xizang **11** female, Xizang. Scale bar: 1 cm.

**Figures 12–15.**
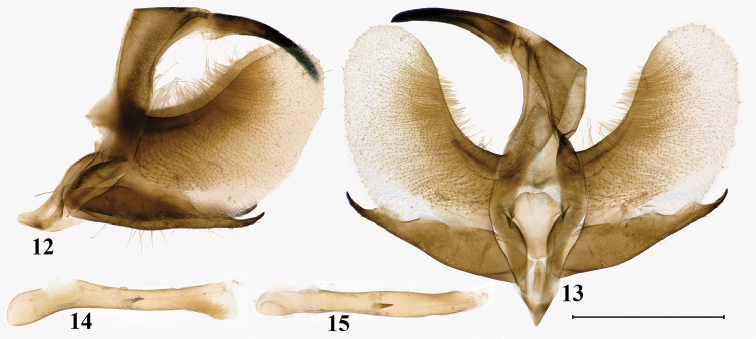
Male genitalia of *Burmeialeesi*: **12** genitalia capsule lateral view **13** genitalia capsule ventral view **14** aedeagus lateral view **15** aedeagus dorsal view. Scale bar: 1 mm.

**Figures 16, 17.**
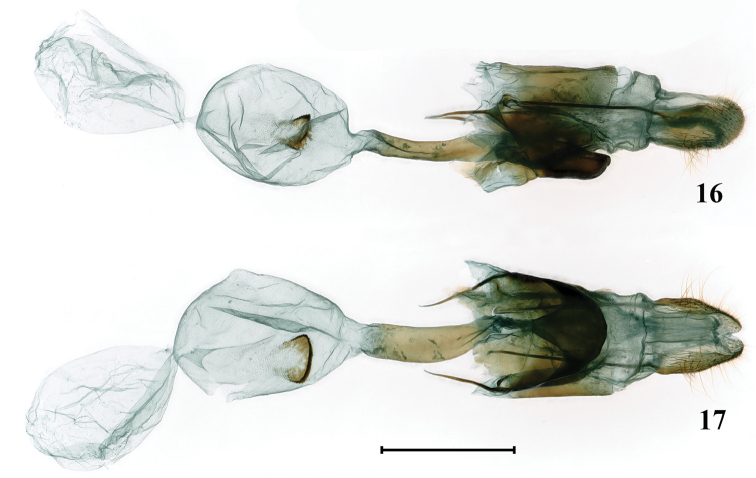
Female genitalia of *Burmeialeesi*: **16** lateral view **17** ventral view. Scale bar: 1 mm.

**Female genitalia** (Figs 16, 17). Papillae anales slightly sclerotized, rectangular in lateral view, with rounded tip. Apophyses posteriores and anteriores slender; the former nearly twice the length of the latter. Antrum membranous and slender. Ostium bursae narrower than antrum. Ductus seminalis short, arising from ductus bursae just below the ostium bursae. Lamella antevaginalis rectangular in lateral view and blunt arrow-shaped in ventral view, strongly sclerotized. Lamella postvaginalis sclerotized and somewhat rectangular in lateral view. Ductus bursae sclerotized, long and broad. Corpus bursae oval, with a strongly sclerotized broad U-shaped signum. Appendix bursae oval and membranous, nearly the same size as corpus bursae.

#### Remarks.

The female genitalia are illustrated here for the first time. This little known species has not been recorded elsewhere after Minet described it in 2003 from northeastern Myanmar.

#### Biology.

**Figures 18, 19.**
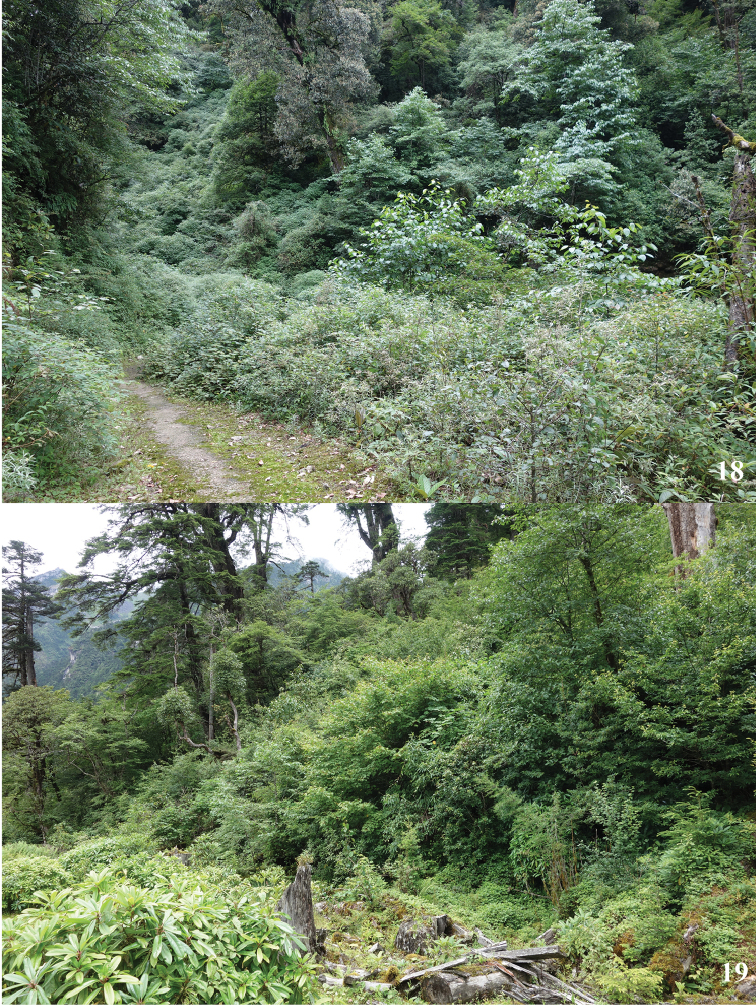
Habitats of *Burmeialeesi*: **18** Yaojiaping, Lushui County **19** 62K, Motuo County.

**Figures 20–24.**
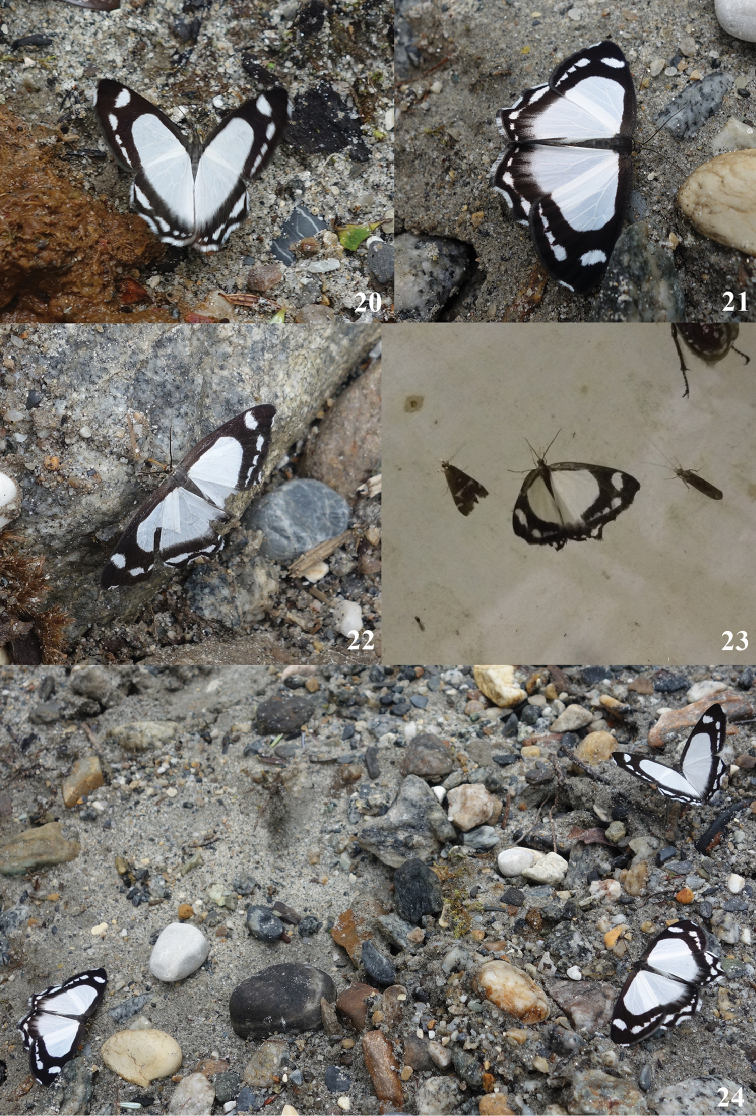
*Burmeia* living adult: **20** sucking on human feces **21** sucking on damp ground **22** sucking on wet stone **23** attracted to light trap **24** gathering and sucking on damp ground. Photographs taken in 62K, Motuo County.

The flying period of this species in China is from early July to late July. Adults are usually found flying slowly above bushes and trees at edge of evergreen broad-leaved forest (Fig. 18) or conifer-broadleaf forest (Fig. 19) in altitude ranging from 2500 m to 3000 m along river or stream. They can fly in both cloudy days and sunny days. Males can be found sucking nutrients on human feces, damp ground, and wet stone (Figs 20–22). In 62K, Motuo County, this species was occasionally attracted to light trap in night (Fig. 23). Sometimes males can gather on damp ground (Fig. 24). In Yaojiaping, Western Yunnan, this species was found flying together with *Psychostrophianymphidiaria* (Oberthür, 1893).

#### Distribution.

China* (Yunnan, Xizang), Myanmar (Htawgaw)

### Psychostrophia

Taxon classificationAnimaliaLepidopteraEpicopeiidae

Butler, 1877

Psychostrophia Butler, 1877: 401, type species: Psychostrophiamelanargia Butler, 1877.

#### Diagnosis.

The genus *Psychostrophia* is characterized by the following characters: hindwing with cilia mostly black, except the part between vein M_1_ and M_3_ white; uncus long, thin and tubular for most of its length; aedeagus with a bunch of slender cornuti and coecum well developed and long.

### Psychostrophia endoi

Taxon classificationAnimaliaLepidopteraEpicopeiidae

Inoue, 1992

**Figures 25, 26.**
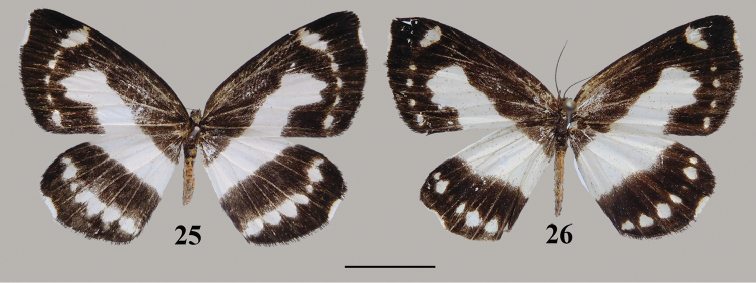
*Psychostrophiaendoi* female, adult: **25** Yunnan **26** Guangxi. Scale bar: 1 cm.

**Figures 27, 28.**
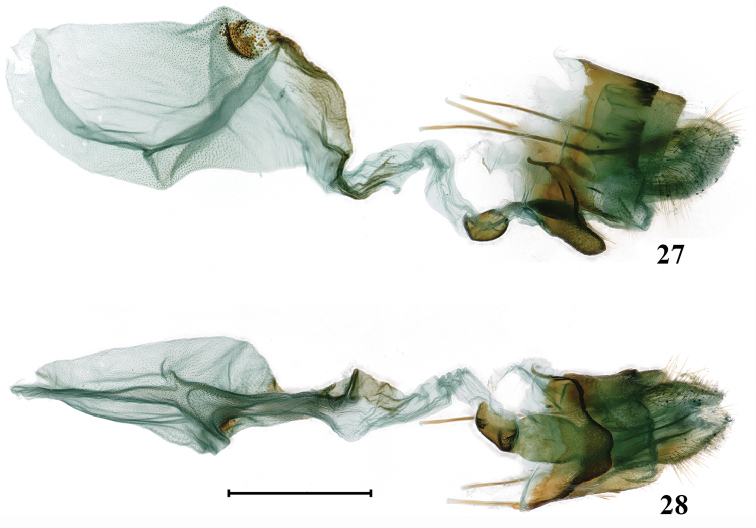
Female genitalia of *Psychostrophiaendoi*: **26** lateral view **27** ventral view. Scale bar: 1 mm.

**Figure 29.**
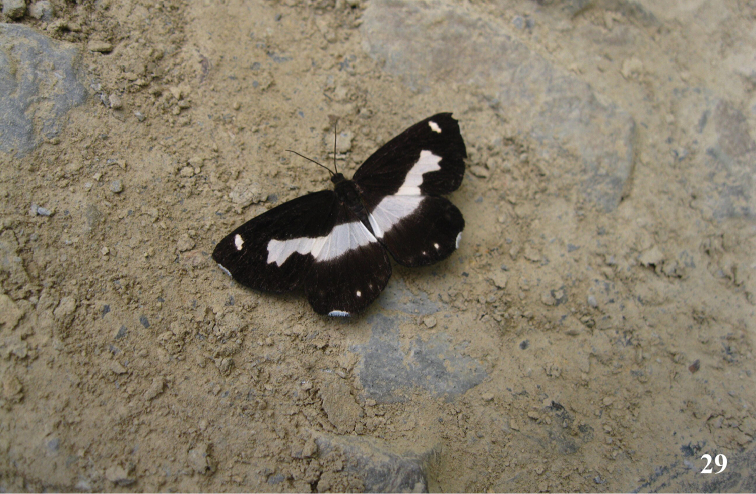
*Psychostrophiaendoi* living male adult, Leishan County, Guizhou Province. Photograph by Gui-qiang Huang.

Figs 25–26 , 27, 28 , 29

Psychostrophia endoi Inoue, 1992: 149, figs 1, 2.

#### Material examined.

1 female, 30.VII.2003, Jiuwanshan Natural Reserve, Rongshui Miao Autonomous County, Liuzhou, Guangxi Zhuang Autonomous Region, leg. Min Wang; 1 female, 9.VI.2014, Deqin County, Diqing Tibetan Autonomous Prefecture, Yunnan Province, leg. Jia-qi Wang.

#### Diagnosis.

*Psychostrophiaendoi* is closely related to *P.picaria* Leech, 1897 from central and western China in external features, but it can be distinguished from it by the following combination of characters: on forewing upper side the postmedian band forming two obviously connected teeth in cell M_3_ and CuA_1_, pointing to termen in both sexes; the submarginal spots absent in male and the submarginal band below the subapical spot weak and represented by separated minute white dots in female; on hindwing upper side the postmedian band is obsolete and represented by white dots in male and the postmedian band ill-developed and represents by separated white dots in female; in male the genitalia valva more protruding at apex; in female genitalia the lamella antevaginalis is trapezoid in ventral view.

#### Description.

Length of forewing 21–22 mm, female differs from male in larger size and having submarginal series on both wings better developed. Head black; antenna filiform, black; forewing ground color black, cilia black from apex to vein R_5_, white from R_5_ to median portion of cell M_1_ and becoming black again from median portion of cell M_1_ to tornus; postmedian band white, extending from middle of the cell M_1_ to dorsum, with its inner edge wavy and outer edge forming two prominent connected tooth in cell M_3_ and CuA_1_; subapical spot white and well-developed, oval shape; submarginal band consisted of four or five separated white dots situated from cell M_1_ to cell CuA_2_; hindwing ground color black, cilia black from apex to vein M_1_, white from M_1_ to median portion of cell M_2_ and becoming black again from median portion of cell M_2_ to tornus; median band white and broad, becoming wider towards costa; postmedian fascia consisted of separated white dots of different size running from apex to tornus.

**Female genitalia** (Figs 27, 28). Papillae anales slightly sclerotized, elliptical in lateral view, with tip rounded. Apophyses posteriores and anteriores slender; and the latter are slightly shorter than the former. Antrum well-developed and forming a strongly sclerotized chamber. Ostium bursae nearly the same width as antrum. Lamella antevaginalis strongly sclerotized, rectangular in lateral view and trapezoid in ventral view, with edge shallowly concave in the middle. Lamella postvaginalis slightly sclerotized and poorly developed, horn-shaped in lateral view. Ductus bursae membranous, long and curved medially, its anterior part near the corpus bursae slightly sclerotized and pigmented. Corpus bursae membranous, large and oval shape, scobinated with numerous small spines; its dorsal ridge near ductus bursae sclerotized and pigmented; next to the sclerotized ridge a sclerotized signum presents, consisting of larger spines.

#### Remarks.

The female of *P.endoi* (Figs 25, 26) is recorded here for the first time. This little known species has not been recorded elsewhere after Inoue described it in 1992 from northern Laos. The differences in the sizes of the submarginal series between females and males (Inoue 1992: fig. 1), due to sexual difference, is rather common.

#### Biology.

**Figure 30.**
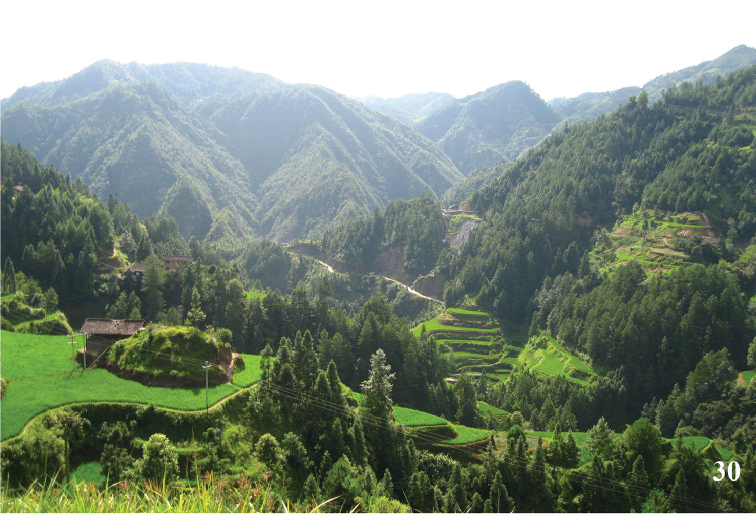
Habitat of *Psychostrophiaendoi* in Leishan County, Guizhou Province. Photograph by Gui-qiang Huang.

The flying period of this species is from early June to late July. The male (Fig. 29) of this species could be found sucking nutrient on wet ground on road of farmlands near evergreen broad-leaved forest (Fig. 30).

#### Distribution.

China*(Yunnan, Guizhou, Guangxi), Laos (Sam Neua).

### Deuveia

Taxon classificationAnimaliaLepidopteraEpicopeiidae

Minet, 2003

Deuveia Minet, 2003: 467, type species: Amanabanghaasi Hering, 1932.

#### Diagnosis.

The genus *Deuveia* is characterized by the following characters: forewing with three thick yellow stripes, juxta bilobate and spoon-like in each lobe and aedeagus with junction piece ventrally.

### Deuveia banghaasi

Taxon classificationAnimaliaLepidopteraEpicopeiidae

(Hering, 1932)

**Figures 31, 32.**
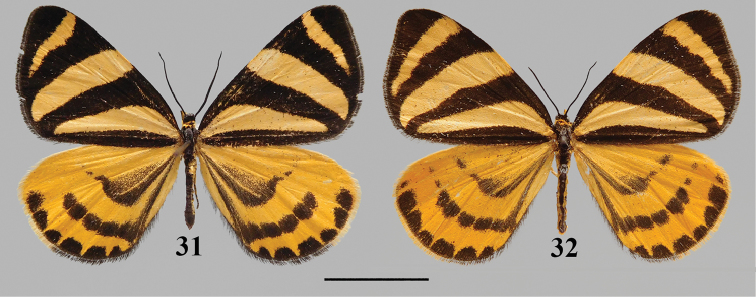
*Deuveiabanghaasi* adult: **31** male, Shaanxi **32** female, Shaanxi. Scale bar: 1 cm.

**Figures 33, 34.**
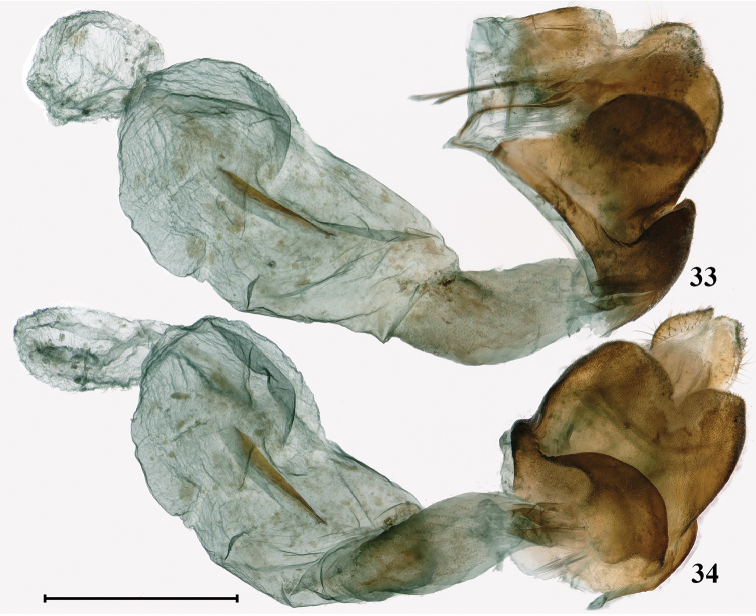
Female genitalia of *Deuveiabanghaasi*: **33** lateral view **34** ventral view. Scale bar: 1 mm.

**Figures 35, 36.**
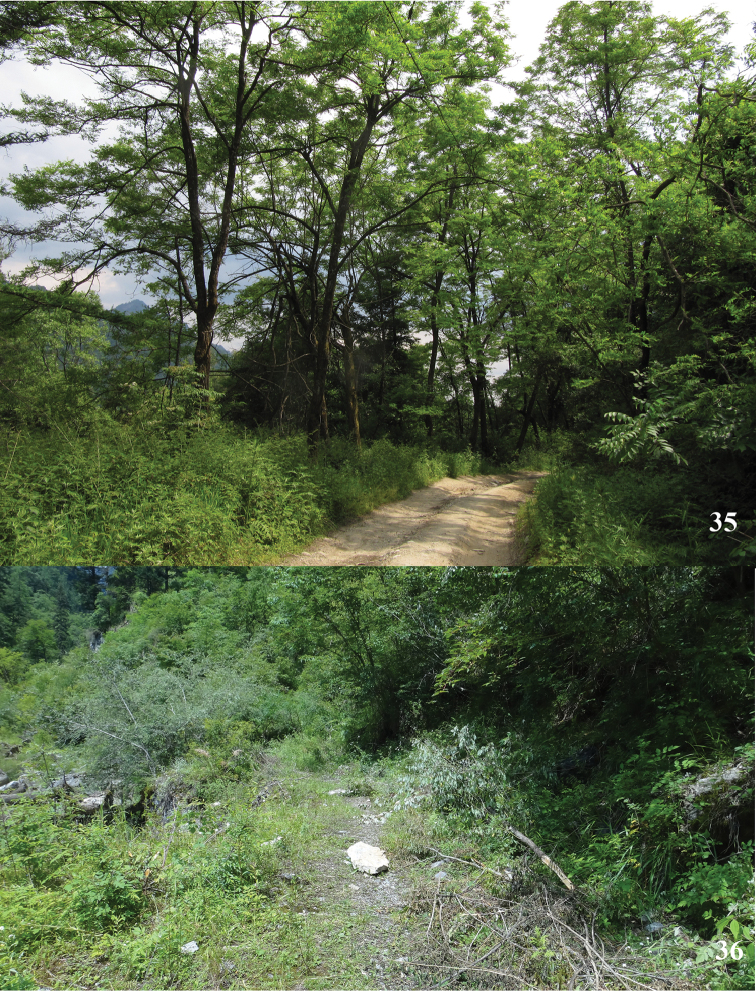
Habitats of *Deuveiabanghaasi*: **35** Yueheping, Ningshan County, photograph by Di Lu **36** Lazikou, Diebu County.

Figs 31–32 , 33, 34 , 35–36

Amana banghaasi Hering, 1932: 28.
Deuveia banghaasi (Hering): Minet 2003: 467, figs 1, 7, 9, 12–17.

#### Material examined.

1 female, altitude 1500–1800 m, 5.VI.2018, Yueheping, Ningshan County, Ankang City, Shaanxi Province, leg. Li-ping Zhou; 1 male, 1400–1600 m, 6.VI.2018, Liuba County, Hanzhong City, Shaanxi Province, leg. Li-ping Zhou; 1 male, altitude 2700 m, 17.VI.2016, Li County, Aba Tibetan and Qiang Autonomous Prefecture, Sichuan Province, leg. Hao Huang; 5 males, 22–24.VI.2017, altitude 2600 m, Pingwu County, Mianyang City, Sichuan Province, leg. Shu-qin Ji; 3 males, altitude 2600 m, 21.VII.2015, Lazikou, Diebu County, Gannan Tibetan Autonomous Prefecture, Gansu Province, leg. Si-yao Huang.

#### Diagnosis.

*Deuveiabanghaasi* is unique among epicopeiid moths for forewing having three broad yellow stripes and hindwing with yellowish ground color and unmistakable black patterns. The female (Fig. 32) is recorded for the first time and a description is given below, the male (Fig. 31) has already been described precisely by Minet (2003).

#### Description.

**Female.** Length of forewing 19 mm, female differs from male in having forewing termen more rounded, postmedian band on forewing upper side broader and longer, and hindwing discoidal cell with less black scale. Forewing ground color black, cilia blackish brown; upper side with three yellowish broad stripes, the basal one extending from wing base across the base of discoidal cell and ending at tornus; the median one extending from middle of the costa and ending at tornus; the postmedian one extending from costa near apex and ending at vein CuA_1_. Hindwing ground color orange yellow, cilia yellow from apex to vein CuA_1_, and black from vein CuA_1_ to dorsum, patterns blackish. Median band extending from vein Sc+R_1_ to distal end of discoidal cell, ending in a long stripe connected to wing base; postmedian band consisted of separated square spots extending from vein Sc+R_1_ to dorsum, and ending in a long stripe connected to wing base. Marginal band consisted of separated rounded spots extending from apex to tornus.

**Female genitalia** (Figs 33, 34). Papillae anales small, slightly sclerotized, rounded in lateral view. Apophyses posteriores moderately long, Apophyses anteriores short and broad, triangular in lateral view. Antrum broad, sclerotized. Ostium bursae narrower than antrum. Lamella antevaginalis strongly sclerotized, triangular in lateral view and broad shield-like in ventral view. Lamella postvaginalis strongly sclerotized, rounded in lateral view and rectangular in ventral view, its edge deeply concave in the middle and forming a V-shaped gap. Ductus bursae slightly sclerotized, short and stout. Corpus bursae membranous, large and oval in lateral view, with a sclerotized blade-like signum in the middle. Appendix bursae presents, globular in lateral view.

#### Biology.

This species is usually found at edge of evergreen broad-leaved forest (Fig. 35) and conifer-broadleaf forest (Fig. 36) in altitude ranging from 1500 m to 3000 m. The flying period is from early June to late July. Adults usually come out around middle of the day when sunshine is abundant and flying slowly.

#### Distribution.

China (Sichuan, Gansu, Shaanxi).

## Supplementary Material

XML Treatment for Mimaporia

XML Treatment for Mimaporia owadai

XML Treatment for Burmeia

XML Treatment for Burmeia leesi

XML Treatment for Psychostrophia

XML Treatment for Psychostrophia endoi

XML Treatment for Deuveia

XML Treatment for Deuveia banghaasi
